# Community Connectors (CCx): the strategies employed by peer to peer connectors to foster relationships with early years caregivers to improve universal early child health and development

**DOI:** 10.1186/s12913-021-06184-y

**Published:** 2021-03-26

**Authors:** C. F. Mills, E. Lowrie, K. Kinloch, E. Hall

**Affiliations:** Blackpool Centre for Early Child Development, (NSPCC), 1 Bickerstaff Square, Talbot Road, Blackpool, FY1 3AH England, UK

**Keywords:** Early years, Child development, Community connectors, Peer support

## Abstract

Blackpool is one of the most deprived Local Authority (LA) areas in England; in April 2015 the Blackpool Better Start (BBS) Partnership was allocated £45 million over 10 years from the Big Lottery Fund (BLF) as one of five ‘A Better Start’ initiative areas in England. The aim of the ‘A Better Start’ initiative is to improve outcomes for children from conception to 3 years of age. Co-designed by professionals and the community, the Community Connector (CCx) programme employs residents to directly engage caregivers of children, in seven of Blackpool’s most socio-economically deprived wards. The CCx follow a socioecological framework which proposes that caregivers will be positively influenced to engage in early years activities because of connections to trained peers. Peer support models are commonly applied within targeted early years health settings (i.e., infant feeding support, literacy) yet their role to improve child outcomes at a universal level has received little attention. This paper focuses on caregiver-level evidence of the strategies employed by CCx - part of an early stage pilot study supported by Frontiers of Innovation, the Harvard Centre on the Developing Child’s Research and Development platform.

The study collated attendance data from Children’s Centres, these are publically funded community centres providing information and activities for families with children 0–5 years of age. The study data included individual interactions between a CCx and caregiver over a 1 year period (1st April 2018 – 31st March 2019). A sampling frame was created from which a total of 22 interviews with caregivers were undertaken in early years community settings. The interview data was thematically analysed; the findings highlighted the mechanisms by which CCx served to mediate service and caregiver communication boundaries, negotiate access to spaces, and encouraged sustained engagement in longer term activities such as volunteering and training. Value was embedded by the CCx in their process of establishing and maintaining connections with caregivers through the ‘everyday’ conversations, their individualised approach and in demonstrating self-efficacy behaviours. Further research is required to review the impact of the CCx role in caregiver’s recall of early years information, nevertheless the study provided important learning for establishing formalised CCx programmes elsewhere, and has implications for community health and early years policy and practice.

**Supplementary Information:**

The online version contains supplementary material available at 10.1186/s12913-021-06184-y.

## Background

The role of communities in the development of the child is well documented, Bronfenbrenner’s Ecological Systems Theory [1] highlights the importance of acknowledging the various ecosystem environments which the child inhabits - this includes the school, home, social communities and the individuals within these- for their development. Children in families with lower incomes are at greater risk of experiencing negative impacts on behavioural, cognitive, social and emotional development [2–4]. The impact of poor health and development within the early years has the potential for life long ramifications across a wide range of domains including: education [5], social mobility [6] and social capital accumulation [7, 8]. The negative impacts can become perpetuating ‘cycles of disadvantage’ for future generations [9].

Blackpool, a coastal resort town in the north-west of England, is the most deprived of 326 Local Authorities (LA) in England – based on average Lower Super Output Area (LSOA) score and concentration of deprivation [10]. In April 2015, the Blackpool Better Start (BBS) partnership was awarded £45 million over 10 years from the Big Lottery Fund (BLF) for their successful application to the ‘A Better Start’ Fund. The aim of the BBS programme is to improve outcomes for children from conception to 3 years of age, in three key areas: language and communication, social and emotional development, and diet and nutrition. Focusing on caregivers in seven of Blackpool’s most socio-econimcally deprived wards: Bloomfield, Talbot, Brunswick, Claremont, Park, Victoria and Clifton (as defined by the English Indices of Deprivation, [11]). The BBS partnership includes the National Society for the Prevention of Cruelty to Children (NSPCC), the Local Authority (LA), Blackpool Teaching Hospitals National Health Service (NHS) Foundations Trust, Blackpool Clinical Commissioning Group (CCG) and representatives from the local community. The BBS partnership is co-ordinated by the Centre for Early Child Development (CECD), the research and development hub of the BBS partnership. The approach seeks to build capabilities (i.e., parenting knowledge, self efficacy, social cohesion) and reduce critical pressures (i.e., alcohol and drug use, poor mental health).

Anecdotal feedback from early BBS consultations with the community suggested that many families in areas of high socio-economic deprivation in Blackpool felt poorly connected and had low engagement with services. This included limited engagement in the publically funded Children’s Centres which provide free parenting advice and activities to families of children under 5 years of age; seven Children’s Centres are located in wards which are part of the BBS programme. Factors contributing to low engagement in services were suggested to include: low trust of professionals, a desire for greater inclusion in the design and delivery of services, and the need for caregivers to have increased agency in supporting their children.

One challenge which perpetuates the impact of deprivation on early child development is the caregiver’s relationship with public services. It has been argued that universally accessible services for young children and their families are necessary to level the social and economic developmental gradient [12, 13] and create equitable conditions for those ‘hardly reached’ [14]. Financial poverty impacts service-seeking behaviour; individuals experiencing less deprivation are more likely to be advantaged in terms of connecting to public services for a range of reasons which centre on a levels of social and cultural capital [15]. Furthermore it is suggested that an individual’s knowledge, ability and access to resources is interwoven with their self-belief of societal positioning alongside peers [16]. Shaped by the discourses of poverty, whether internalised by the individual or through external media, there are a range of factors which may influence how those experiencing poverty communicate with services and contribute to disengagement i.e., feelings of shame, stigma, low self-esteem, a perceived lack of power and insecurities [8].

There is increasing interest in peer to peer models which seek to bridge the gap between the community and professionals/services [17, 18]. Within health literature there are numerous labels for individuals that seek to ‘boundary span’- encourage communication between individuals or groups within society that may be experiencing a metaphorical barrier [19]. Awarded various titles: health champions, community navigators, community connectors, the roles share commonalities: the involvement of those from within, or have familiarity with the communities in which they operate [20], and the provision of education or direction to resources in a specific subject area [21]. These roles are suggested to contribute to positive behavioral change due to their ability to foster connections through ‘lived experience’ and local knowledge of the community in which they are located [22]. It is proposed that such ‘connectors’ provide, “an effective, flexible, relational and localised response that can improve service access and health outcomes for people who are traditionally hardly reached” ([18], p.372).

There is an increasing propensity for Clinical Commissioning Groups (CCGs) and health funding bodies to utilise Community Connectors programmes, recognizing the potential positive health implications for shaping lifestyle decisions and enhancing local capacity of services [22]. The criterion for such ‘Connector’ programmes vary from age-related support programmes (e.g. AgeUK Camden Community Connectors), to universal sign posting to generic health services (e.g. Powys Association of Voluntary Organisations Community Connectors). Other services blend the peer to peer model with social prescribing in which referral to these connectors are instigated by General Practitioners (GPs) and/or health care practitioners (e.g. British Red Cross Connectors programme which seeks to address loneliness and social isolation). Similarly, there is a wide array of literature exemplifying formalised peer-support models to support positive health behaviour change through targeted messaging including increasing physical activity and reducing smoking [23].

The peer to peer approach has been applied to targeted services within the early years, such as infant feeding practices [24–26], parental mental health support programmes [27, 28], and for caregivers of children with additional needs [29, 30]. But as yet there are no documented approaches to the integration of peer to peer support to improve early child health and development outcomes at a universal level which is why this study is important.

### The Community Connector (CCx) programme

The inception of the BBS Community Connector (CCx) programme began in 2016. One area identified during the initial consultations for BBS was caregiver’s awareness of, and access, to locally based early years services. A representative group of local caregivers, ‘Community Voice’, was formed to support the design and development of BBS programmes. The group advocated employing locally trained peers who were suggested to have ‘lived experience’ of being caregivers in Blackpool to engage with caregivers, provide early years information, and encourage participation in activities. The CCx programme was designed for local residents to directly engage caregivers of 0–3-year-old children in the seven identified BBS wards. In designing the role the working hours, qualification criteria and locations were adapted to ensure it was accessible to a wide range of individuals from the target area. The primary focus was on individual motivation and local knowledge of early years. Each member of staff employed as a CCx receives university-accredited training in outreach and detached methods of working. The CCx are trained to develop ‘primary’ connections with local families to: i.) deliver key messages around early child health and development, and ii.) encourage participation in early years activities. In turn, the caregivers may be influential in positive behavior change within their own communities through a contagion model which suggests their actions may be mirrored by others within their social networks; this model can increase the reach of public health interventions to a wider population, and improve sustainability [26].

The CCx programme aims to reduce stressors and build capabilities of early years caregivers, by supporting them to actively identify and address their own and their child’s needs; building awareness of, and participation in, early years activities; as well as encouraging families to become more involved in local decision through participation in the Parents Forums of Children’s Centres. The aims are supported by an increased level of perceived authenticity and trust derived from the lived experience and location of the individual CCx within the caregiver’s communities. Utilising the Centre for Early Child Development (CECD) community development framework of Reach, Engagement, Change: Reach – to appeal to a universal audience but specifically those ‘hardly reached’ who have not yet accessed early years activities, Engagement – to encourage and support these caregivers to participate in activities and Change – to contribute to positive behavior changes. The CCx programme was established for ‘Reach’, being responsive to individual caregiver needs, to support engagement in appropriate activities. There is no prescribed structure for the CCx in terms of establishing the primary connection with caregivers.

In December 2018, the CCx programme was submitted to, and accepted into, the Frontier of Innovation (FOI) portfolio - the Center on the Developing Child [31] at Harvard University Research and Development platform. The FOI aims to accelerate the development and adoption of science-based innovations that achieve breakthrough impact at scale. It is a diverse portfolio of new, innovative, testable services or programmes which have the potential to transform the lives of children and families facing adversity. The CCx programme is the first project to be accepted into this portfolio from the UK. This 1 year project allowed researchers from the CECD, which is responsible for the strategic planning and delivery of BBS programme, the opportunity to collaborate with researchers from Harvard University implementing the IDEAS Impact Framework™. The IDEAS Impact Framework™ is a rigorous process for developing, evaluating, and iterating programs or intervention strategies, with the goal of supporting teams and organizations to achieve significantly greater impact on the life outcomes of children and families at a population level. It draws on existing research and development tools, applying them in new ways to set a higher bar for programme development and evaluation. The IDEAS Impact Framework™, is a joint initiative of the Centre on the Developing Child at Harvard University, the University of Oregon Centre for Translational Science, and the University of Washington College of Education. IDEAS is an acronym for; Innovate to solve unmet challenges; Develop a usable programme with a clear and precise theory of change; Evaluate a theory of change to determine what works for whom and why; Adapt in rapid cycle iterations and; Scale promising programmes.

The CCx programme was submitted to the FOI portfolio as an early stage pilot study focused on early implementation. The evaluation of formalized CCx programmes often measure the subjective wellbeing of the participant from initial engagement in a service to the end of the intervention period (e.g., [32, 33]). In contrast, the BBS CCx programme is designed to be dynamic and respond to individual need. Unlike other approaches involving formalised CCx there is no standardised intervention period or manualised strategy for the CCx engagement with a caregiver. It was acknowledged that greater understanding of the strategies used by CCx would enhance any subsequent manualisation of the programme. This is important for future work as manualisation is key to understanding fidelity [34] allowing consideration of how variations in delivery may influence outcomes for participants. Only by understanding how a programme is implemented is it possible to record the strategies and processes which contribute to the outcomes experienced by the caregivers. The learning from the CCx programme evaluation is important for the development of effective local services, and for wider community health and early years policy and practice, awareness of the enabling and process factors which may influence the reception of messages using peer to peer models.

### Influences in the study

At the time of the study (1st April 2018 – 31st March 2019) each CCx was based in a Children’s Centre within each of the BBS wards, however these Council-run Children’s Centres were undergoing a service review. The review saw the creation of a ‘hub and spoke’ model which involved increasing the capacity and range of family services at three Children Centres (‘hubs’), whilst reducing access hours and services at four Children’s Centres (‘spokes’). This potential impact on access to spaces was noted by some of the caregivers during interviews.

## Methods

### Design

Evaluations of Community Connector programmes often measure the subjective wellbeing of the participant from initial engagement in a service to the end of the intervention period (e.g. [32, 33]). In contrast, the BBS CCx programme is designed to be dynamic and respond to individual need, there is no standardised intervention period or detailed strategy for their connection with caregivers. It was important for the study to identify the CCx strategies which contribute to the outcomes experienced by the caregivers as it would permit simple manualisation of the programme. The evaluation involved i.) creating a sample framework using Children’s Centre database records of the number of individual caregiver contact with a CCx, ii.) semi-structured interviews with a random sample of caregivers selected from the Children’s Centre sample framework, and iii.) demographic information from caregivers that participated in the semi-structured interviews. The report is written in line with the consolidated criteria for reporting qualitative research (COREQ) [35].

#### Children’s Centre data

Each Children’s Centre records individual access to their premises, including attendance at Children Centre and BBS-led universal activities e.g. BBS community consultations and collates registers, provided by the CCx, of their contact with caregivers. Annonymised data was provided by the Children’s Centre for a 1 year time period, 1st April 2018 – 31st March 2019. The data was used to identify the number of contacts each CCx had made with each individual caregiver, this was used to create a sampling framework for the recruitment of caregivers for interviews. The database showed that 493 unique individuals that had an interaction with a CCx during the 1 year period. The records of when a CCx had interacted with a caregiver also illustrated caregiver engagement in activities pre and post CCx interaction.

#### Semi - structured interviews

The researcher undertook semi-structured interviews with the caregivers selected from the sample framework created from the Children’s Centre data. All participants were asked for consent for the interviews to be audio recorded. The interview guide which was created for the study (Additional file 1) covered three keys areas: a) exploring the strategies employed by each CCx in their ‘primary connection’, b.) exploring the activities/resources that caregivers were directed to by a CCx and, c.) any change which the caregiver experienced which they may attribute to their interaction with the CCx. Whilst an interview guide was used there was scope within the semi-structured interviews for the caregivers to identify areas not included in the guide. To aid conversation around the caregiver’s participation in early years activities, a prompt sheet listing all potential Children’s Centre and BBS activities with additional categories of community/faith based groups/activities and ‘other’ was used. Notes were made on these during the interview to identify the participant’s response.

The interviews were undertaken over a three-week period and held in private rooms, with only the researcher and participant present, at child-friendly community venues local to the participant, thus permitting caregivers to be accompanied by their child/ren.. Interviews lasted 25 to 55 min, this was dependent on individual’s responses, influenced by their recollections of the interaction with a CCx. All participants were provided with a written consent form and participant information sheet which detailed: the purpose of the research, requirements of the participants, use and storage of the data, confidentiality, consent, voluntary nature of the evaluation, right to withdraw and contact information for the study. These were verbally explained prior to the start of the interview. At the start participants were also asked to complete basic socio-demographic information.

#### Demographic information

Demographic information for interview participants was collected by the researcher at the start of each interview, participant’s were asked to provide their ward of residence, length of residence in Blackpool, age, number and age of children, employment and relationship status to provide context for the reach of the CCx programme.

### Data collection

The target participants for the study were Blackpool caregivers who had one or more interactions recorded with a CCx over a 12-month period, from 1st April 2018 to 31st March 2019, on the Children’s Centre database.

#### Sampling

A sampling frame was created in which each caregiver, referenced by an anonymised identification number, was grouped by i.) the number of contacts they had with the CCx: 1 contact, 2 to 4 contacts, or 5+ contacts, and ii.) the period of time over which these contacts occurred: 1 week, 1 month (30 days), 1 to 3 months, 3 to 6 months or greater than 6 months. This led to 15 categories. A random sample of seven caregivers was then selected from each category by entering the identification labels into a random number generator, if there were less than this number of participants in a category then the entire category would be selected. The number of participants was selected with the expectation of achieving 20–30 interviews, aligning with the capacity and time limited nature of the study. Two anomalies were noted in which the number of contacts between a caregiver and a CCx was significantly greater (with over 60 contacts over a 1 year period) than the observed trend. These two participants were purposively selected to be invited for participation in the study.

Individual identification numbers were sent to relevant Children’s Centre staff to contact families to gain consent for the research team to contact them regarding participation in the study. A guidance brief and conversation template were provided to the Children’s Centres to support this process. In total 95 individual identification numbers were provided to the Children’s Centres. Challenges with recruitment identified by Children’s Centre staff included: incorrect/incomplete contact details on the database, no response after multiple attempts to contact or no recollection of an interaction with a CCx. Those who provided verbal consent to contact to the Children’s Centre staff were contacted by phone by the researcher who explained the study. At this stage 25 of the 30 individuals that could be contacted by the Children’s Centres agreed to an interview, however three individuals at the interview stage then indicated no recollection of contact with a CCx. It was suggested that this may have been impacted by a combination of a limited number of contacts (1–3) and the length of time over which contact occurred (3–6 months, 6 months plus). Similarly recall may have been impacted by the space or activity in which any conversations occurred. In total, 22 interviews (Table 1) were undertaken.

**Table 1:** Number of individuals interviewed shown by number of CCx contacts and time over which this occurred

Period over which contact occurred	Number of CCx recorded contacts		
	1	2–4	5+
**1 week**	1	2	
**1 month**		2	1
**1–3 months**		1	1
**3–6 months**		4	4
**6 months +**		1	5

#### Demographic characteristics

Participants were aged between 25 and 49 years, the mean age was 32.4 years. There was only one male caregiver in the sample. Of the 22 caregivers interviewed, 17 resided in BBS wards, and the remainder lived in Blackpool but outside of the BBS target wards, potentially highlighting the movement of caregivers between Children’s Centres. Most caregivers stated that they had lived within Blackpool (this included internal transience between one or more wards) for over 10 years (n14). Of the remainder, five had been resident in the area for between 5 and 9 years, two for 2–4 years, and only one individual had lived in the area for less than a year. Despite the length of residence in the town, for most participants this did not appear to increase their likelihood of engaging with early years activities.

Only one individual identified themselves as a single parent, and the remainder identified that they were in a relationship (including marriage), there was no further explanation as to whether the relationship was with the parent of the child, or status of the relationship. Most caregivers had two children (13n), although four identified having four or more. The number of participants currently with a child aged 0–3 years was 15, this increased to 19 if it was broadened to include children up to 5 years of age, to allow for contact made by a CCx during the last year. This highlights that connections had been recorded with caregivers (3n) whose child/ren were no longer within the criterion for BBS.

Employment characteristics were wide-ranging and included: unemployed (3n), full time care-givers (5n), employed part time (7n), working full time (2n), students/studying (2n) and declared long-term sickness/disability (2n).

### Analysis

All interviews with caregivers were audio recorded and transcribed. The transcribed interviews were entered into NVivo 12 analysis software for thematic coding. The data was coded by inductive and deductive approaches, using the interview schedule to group the responses, further to which the nuances of the conversations were identified by thematic analysis [36]. The codes were reviewed by three different members of the research team.

In the reporting of the study all participants were given gender neutral pseudonyms; with regard to the staff employed as CCx, all references to he/she have been adapted to them/they to minimise risk of identification. Gender of the CCx was not integral to the aim of the work, nor was it raised in discussions from participants.

## Findings

The paper focuses on caregiver-level evidence of the strategies employed by the CCx in establishing primary connections with caregivers and the extent to which this ‘connection’ may impact parent stress and self-efficacy, contributing to the individual’s awareness of, and participation in, early years activities/learning and perceived social/community support.

The following three overarching themes which arose from analysis of the findings are presented for the reader: the positioning of the CCx; their knowledge of the individual caregiver, and the CCx demonstrating behaviours to promote self-efficacy. Whilst they are presented here as separate sections it should be recognised that these themes are interwoven; it is not possible to suggest the degree to which each must occur to create the connections which the caregivers described.

### Positioning of the CCx

The CCx programme sought to position the CCx as peers for the caregivers, with ‘lived experience’ of the local area. Geographically, CCx were located ‘in’ the community using early years settings, often Children’s Centres, in neighbouring wards to those areas in which they resided.

Geographically the presence of CCx, predominantly within local Children’s Centres, appeared to reinforce the caregiver association of their role with this space,

“I was always under the impression that they were like to do with here … So, obviously they are part of Better Start and, well, not just what’s going on here, but at different Children’s Centres.” (P17)

Although the majority of caregivers suggested a wider remit of the CCx in encouraging “… involvement in the community” (P12), ultimately, perceptions of their role centred on drawing families into the centres,

“They’re trying to get more people involved in coming to Children’s Centres and making sure they’re aware of all the facilities and things that are available.” (P15)

The CCx prescence in these settings was suggested to create familiarity and for some this regular contact conferred trust, as one caregiver commented, “I could trust quite a few of them because obviously being there all the time.” (P7). Alongside which, the welcoming behaviours demonstrated by the CCx appeared to instill confidence in the caregiver to be themselves in the space, inferring that it was their space, “You’d just pop in for a coffee and then I felt quite myself, to be fair.” (P7). Some caregiver’s suggested that this was associated with having a shared experience with the CCx,

“They make you feel like you would be welcome, you’re not the only one in that boat sort of thing...” (P16)

Nearly half of the participants inferred ‘existence value’ from knowing of the presence of CCx in the early years setting, “I think it’s nice to know that you can come in and talk to them …” (P20). The importance of this ‘local’ presence in an accessible space was also noted by caregivers in relation to their concerns of reduced accessed to Centres from the Children’s Centre review,

“You need those friends, you do, and them closing all the Children’s Centres and stuff, I think especially parents like me who have got no bloody money to pay to go places.” (P22)

Conversely, the location of the CCx within Children’s Centres, which helped to evoke familiarity for some, may infer that the CCx were interacting with those individuals who already accessed these spaces, rather than engaging with caregivers deemed to be ‘hardly reached’. Although anecdotal feedback from Children Centre staff suggested that whilst many caregivers may access the social ‘café’ spaces in the Centres, this type of access did not indicate engagement in early years activities. This was echoed by several caregivers who acknowledged that they would visit the Children’s Centres to sit in the café.

Caregivers acknowledged accessing early years information through other sources, including the Children’s Centre staff and social media pages, however the CCx programme appeared to offer a distinct approach by creating personal connections,

“It’s more that there’s friendship there as well, although I’ve known the Children’s Centre manager longer, it’s not on a personal level as well, like I don’t know if the Children Centre manager knows all my children’s names, you know?” (P20)

Many caregivers supported this stance, positioning the CCx between a friend and a professional which appeared to confer acceptance of the CCx as a peer,

“It’s half half, 50:50 friendship and 50 business, if you’d call it business, I don’t know.” (P15)

One explanation for this is the variable location and timing of interactions between the CCx and caregiver. Although all bar one of the primary connections had occurred in Children’s Centres, several caregivers remarked on how they had subsequently been acknowledged by the CCx in non-work locations. It was intimated that this was something caregivers did not experience with other workers,

“Drew doesn’t blank them like some, some people they do, it’s that work and home life, they stop it, Drew doesn’t, Sam doesn’t, so to me they’re still working around the clock when they’re not working, because they’re still talking to families and everything …” (P5)

In the longer term, the relational and geographical positioning of the CCx appeared to encourage the caregiver to move between different/unfamiliar spaces. For some caregivers this encouragement to participate in groups led to reduced feelings of isolation, “It helped me not be so lonely whereas I would have just sat at home and slept.” (P15). Further to which two caregivers acknowledged the positive impact for them in periods of stress,

“… It’s like the day stress when it’s like my kids stress me out or if they’ve done something and I need a bit of advice that could help with that or suggest somethings I could do” (P7).

Whilst others reported on having support to negotiate access to other community spaces, two individuals commented on the support they received to utilise other community venues to establish early years activity groups. Yet there were several comments from caregivers which intimated reliance on the individual CCx,

“… because you can ask for more and they can try and get it, it’s there on tap, constantly whenever you need it, it will be there” (P5).

The positioning of the CCx as providing *to* families was echoed by several caregivers who associated the role of the CCx with the wider community, to encourage involvement *of* and *in* the communities of the town,

“… They’re just trying to get everyone together and get everyone out and enjoying the town that we have …” (P16)

“To provide information about activities for families due to their knowledge of “everything that’s going on” (P20).

Whilst some caregivers indicated awareness of the time limited nature of the role, as one remarked,

“I would like them to be there at all times though! I know that their budget is going to run out soon and obviously we’re gonna be like ahhhh, they’re a bit of a security blanket for us at the minute” (P14).

It is apparent that further consideration should be given to the potential for caregivers to become reliant on their relationship with the CCx as providing for them and approaches to increase their connectivity to peers within their community.

### Knowledge of the individual

Communication strategies for CCx interactions centred on personalised knowledge of the caregiver, holding the everyday conversation, and perceptions of the immediacy of their response to caregivers.

Over half of the caregivers commented on how the CCx would exhibit interest in them as individuals. Caregivers referred to the CCx recalling personal events or making informal enquiries about aspects of their life they had discussed such as holidays, college courses or general health. The personalised communication displayed by CCx contributed to feelings of trust and presented a genuine interest in the individual.

“It’s like all of them, it’s the way they are, they way they do it, they’re friendly and they listen, you know they do because they suggest things. Things that are for you and [they] work out what you need … They’re always trying to get the best out of you and offer you different ideas” (P13).

This reaffirmed the relational positioning of the CCx to the caregiver; located between a friend and professional, discussed in the previous section. In this way, the CCx was viewed as a peer, whether through revealing their own experiences with their children, or through their visual presence outside of their role within the local communities. For one respondent, the trust in the CCx and the value placed on that relationship was such that they felt a need to confide in the CCx about a significant life event prior to any of their family or friends,

“They were one of the first people I told … I was supposed to be doing something and I just didn’t want to do it … it sounds like a copout, so I just opened up.” (P15).

Demonstrating interest in the individual was also exemplified by the CCx strategy of holding the everyday conversations, enquiries of ‘how are you doing?’ were referred to by many of the caregivers, with CCx following through on responses,

“If Jamie comes past you and says, ‘you alright?’ You say ‘No, I’m having a rubbish day today’ … then Jamie’ll do something or say something and it’s like that door’s open …” (P5).

This metaphorical ‘door opening’ highlighted by the caregiver, is an important aspect of linking the caregiver to resources and may lead to further directed conversation. One further way in which this was achieved, pertinent to the early years, and cited by numerous caregivers was the direct interaction sought by the CCx with the caregiver’s child/ren, whether this was acknowledging them ‘in the street’ or in the Children’s Centres, the importance of this was recognised by several caregivers,

“When you’ve got young children it’s not just about you, it’s about your children so it’s important they can talk to them …” (P20).

Responsiveness of the CCx was also an important trait; this was regarding availability of information, as described in the previous section it was viewed as ‘on tap’, this was also described in terms of the breadth of information available. Several caregivers remarked that the CCx had indicated they would seek to answer any question,

“Riley was ‘Just call me, any questions, and if I don’t answer send me a text message and as soon as I pick it up I’ll get back to you ‘” (P15).

Additionally, the CCx would seek to maintain the contact with the caregiver through texts,

“They’ll send a text to remind me, because I forget things, it’s just that keeping in touch” (A13).

The repeated interactions were important in maintaining and reinforcing the relationship as peers, through using modes of contact i.e. text messages, that are more likely to be used in social rather than professional interactions.

### Behaviours promoting self efficacy

The CCx programme appeared to contribute to the self efficacy of the participants through the behaviours they employed when interacting with caregivers, this included verbal encouragement, modelling behaviours and vicarious experiences.

Over half of caregivers referred to the CCx encouraging additional participation in longer term activities such as training courses or early years volunteering activities. Many caregivers suggested this was due to the verbal encouragement of the CCx,

“I wouldn’t have been doing anything to be honest that I was doing. Without Toni’s encouragement, I wouldn’t have come in.” (P11).

The verbal encouragement was reinforced with practical support of the CCx at groups, such as at Children’s Centres Parents Forums, these forums help to shape the activities and services at the Centres. It was suggested that the CCx provided advice and encouragement that resulted in some parent’s committing to the groups, one caregiver remarked,

“I would have ended up dropping out of the parent’s forum probably pretty quick had Jamie not been there *…* if I hadn’t had that support I probably, definitely, would have said, ‘look, I’m not, I don’t know what I’m doing so I’m not doing it any more’ and I would have just walked away and left …” (P15).

The CCx was suggested to model proactive behaviours, for example in encouraging peer to peer interactions,

“Like they’ll say, ‘Oh such and such, she’s doing the same, why don’t you have a chat’ … if they see someone like sat on their own, they’ll say ‘Oh I know who you can speak to’ and they’ll bring them over’” (P6).

In such a way, the CCx demonstrates how to instigate social connections, linking caregivers within their communities through an apparent authencity which is derived from their knowledge of the caregiver. The process of seeking to directly link families to other families was remarked upon by one caregiver who recalled observing a CCx interaction,

“We was on the park actually and they came over with a family, I don’t think they [the family] lived here long and Flynn was showing them around … introduced them to Nic [another caregiver] and stuff, and now this guy and Nic, now they’re friends and they’re going to do the Forest school and stuff together” (P21).

The potential impact of such behaviour may stimulate friendships, as observed by the caregiver, or reinforce existing connections by instigating action of the caregiver,

“It was the connector saying, ‘Make sure you tell everyone about us. Make sure you get whoever wants to come, make sure they can’ So that made me like then go and get them to come” (P12).

It was also suggested that such behaviour may contribute to confidence for caregivers to undertake these actions independently, “I know more about who to speak to myself and I feel I can go up and ask them” (P5).

The CCx programme appeared to contribute to the self efficacy of the participants by the interrelated areas of their positioning and communication, including verbal persuasion and modelling proactive behaviours. These actions could lead to a positive reinforcement cycle whereby the encouragement of the CCx leads to engagement in activities, which subsequently confirm the positive messages of the CCx. One caregiver explained how the CCx encouragement had impacted their feelings of anxiety,

“You’re not the only one in that boat sort of thing... I was quite anxious about going and I was going to use the weather as an excuse not to go...I’ve been struggling to get out of the house with everything’s that’s happened after having [the baby] and that, so I just feel a bit overly anxious I think. But I thought ‘sod it, we’ll go’ but everyone was just so nice and friendly and you walk past them and they’re, ‘Hi are you alright?’ Are you having fun?’ and it’s just, well this wasn’t too bad! I’m glad I came” (P16).

The culmination of behaviours demonstrated by the CCx may contribute to vicarious learning for caregivers and may have contributed to a heightened self awareness of information or resources that they don’t currently possess,

“Charlie feeds back the information we don’t have and Charlie reaches people that we can’t reach and has the experience that we don’t have, so like, until we have that experience then we need Charlie on board …” (P17).

It was suggested that for the current time the CCx “… putting training and stuff on with us and putting us on the right paths and stuff …” (P14). This infers both an acceptance of the CCx having the ‘right’ information but ultimately may serve to reinforce that as peers in their community it is something they can also attain.

## Discussion

The CCx programme early stage pilot study was undertaken as part of the FOI portfolio, and aimed to identify strategies employed by the CCx in establishing connections to early years caregivers. Unlike existing programmes which often measure the subjective wellbeing of the participant from initial engagement to the end of the intervention period (e.g., [32, 33]), the BBS CCx programme is flexible to individual need, there is no standardised intervention period.

The study suggested that for these caregivers the CCx programme provided a locally based, flexible ‘boundary spanner’ (see [18]), building upon the existing literature this study explored the nuances within the process of the CCx establishing connections with caregivers. The findings suggest that the interwoven strategies: the geographical and relational positioning of the CCx, their knowledge and recollections of personal information and subtle demonstrations of self-efficacy behaviours helped to address some of the explanations for individual’s disengagement in services reported in literature i.e., low self-esteem, a perceived lack of power and insecurities [8]. Whilst it may be suggested that the actions of the CCx helped to reframe caregiver perceptions of professionals, which contributes to low interaction with services [8, 15], this area was not explored further in this study.

In the study sample of caregivers, ‘Reach’, in terms of the Reach, Engagement and Change, the BBS approach of community development, had already been achieved - all bar one of the caregivers interviewed were accessing their local Children’s Centre prior to their interaction with the CCx. It is suggested that the caregiver’s level and type of engagement was positively influenced by the CCx, over half of the participants suggested the interactions had contributed to engagement in longer term activities such as volunteering or training. For others, the impact of the interaction with the CCx extended to participation in activities or events in unfamiliar spaces that they may have been anxious about attending on their own.

The positioning of the CCx within their communities as a worker and community member may also serve to affirm the CCx position as a peer. The modelling behaviour of CCx when not in their work role and the informal interactions with caregivers may help the delivery of targeted messages. In such a way caregivers may view information as being delivered from an aunthentic position of a trust rather than with any predesigned agenda. Whilst there was limited recollection by caregivers of directly being informed of early years messages – the majority of those interviewed suggested that the caregiver’s role was to encourage community involvement in the town – it was inferred from the interviews that early years messages were delivered indirectly by the CCx interactions. This could occur, for example, in the CCx demonstrating behaviour through their positive interactions with the caregiver’s children, in role modelling initial interactions with other caregivers to instigate peer to peer connections, or by encouraging longer term engagement in early years activities that may encourage engagement with formal skills and learning for the caregiver.

The learning from the CCx programme study is important both locally in terms of practical development and implementation of effective services, and for wider community health and early years policy and practice. While the value of peer to peer ‘connector’ relationships in health services is widely documented [17, 18], the study encourages awareness of the enabling and process factors which may influence the reception of universal early years messages using peer to peer models. It highlights the challenges for the staff employed as CCx in negotiating this position, as both a peer and a professional. The peer approach of the CCx is both an opportunity and challenge, it is a delicate balance in applying the strategies of a peer within the defined boundaries of a programme and a paid role. It is widely acknowledged that the trust within peer support approaches positively impacts retention [17, 37] however in this instance, the CCx are not seeking to retain caregivers in the CCx service but to forge those outward connections to other groups and community members. One potential concern is encouraging reliance on the CCx, some caregivers referred to the CCx being ‘on tap’ (P5) or a ‘security blanket’ (P14). This was also echoed within references of the CCx role in providing *to* or *for* the families, as some caregivers positioned the CCx as having knowledge or experience which they did not yet have. Therefore there is less onus on the caregiver to develop their own social connections or acquire the skills and/or confidence to independentally interact with services.

## Limitations

The analysis and interpretation of qualitative data is inherently subjective [38] despite attempts for objectivity *‘ordinary agents are always ‘inside’ a social world that encompasses them’* ([39], p.4). The social constructivist perspective recognises that external (e.g., culture, society, beliefs, experience etc.,) and internal (e.g., physical) factors shape individual interpretation of meaning. Furthermore, challenges of the evaluation lay in the ability of caregivers to articulate the impact of the CCx. It was acknowledged through the analysis of the interviews that values for caregivers were interwoven within the behaviours and strategies the CCx employed during interactions. It is important to recognise that the complexity of these interwoven behaviours shaped the interpretation and presentation of the findings, any attempt to distinguish independent sections may be seen to undermine the importance of each contribution to the whole.

## Conclusion

There is increasing interest in the role of CCx programmes in helping to connect those who are ‘hardly reached’ to Public Health services and information. The CECD CCx programme is unique in employing CCx for the target audience of early years caregivers. Value for caregivers was embedded in the CCx process of establishing and maintaining connections through the interwoven strategies of geographical and relational positioning, their individualised approach and in demonstrating self-efficacy behaviours. This encouraged peer to peer relationships, developed service seeking behavior with the CCx and contributed to self-efficacy by role modelling and verbal persuasion. Whilst it was not possible to determine changes in the knowledge of early years by the caregivers, it is suggested that sustained engagement in longer term activities (i.e., volunteering in early years settings) may ultimately contribute to increased learning and skill development.

The fast cycle iteration undertaken from implementing the Harvard University IDEAS Impact Framework™ led to several immediate actions including: reviewing the access of CCx staff to update caregiver contact records to aid reliability, increased presence at early years community activities, creation of individual CCx staff contact cards to increase recognition in the community, single message delivery campaigns and simple manualisation of the programme. Further work is needed which considers recall of early years message delivery using the peer to peer model, and the extent to which sustainable relationship exit pathways may be developed, nevertheless the work contributes to wider learning regarding implementation of formalised community connector roles for health policy and practice.

## Supplementary Information

**Additional file 1.** Interview Guide.

## Data Availability

Data sharing is not applicable for the qualitative data transcripts. There was no expectation for the raw data transcriptions to be shared and as such the ethical application and consent process did not include permission to share this material.

## Abbreviations

BBS: Blackpool Better Start
BLF: Big Lottery Fund
CCG: Clinical Commissioning Groups
CCx: Community Connector
CECD: Centre for Early Child Development
FOI: Frontiers of Innovation
GP: General Practitioner
LA: Local Authority
LSOA: Lower Super Output Area
MHCLG: Ministry of Housing, Communities and Local Government
NHS: National Health Service
NSPCC: National Society for the Prevention of Cruelty to Children
REC: Research Ethics Committee
ToC: Theory of Change
