# Supplementary material for: Community Connectors (CCx): the strategies employed by peer to peer connectors to foster relationships with early years caregivers to improve universal early child health and development
Source: BMC Health Serv Res. 2021 Mar 26;21:283. doi: 10.1186/s12913-021-06184-y (PMC8004447; doi:10.1186/s12913-021-06184-y)
Supplement: Supplementary file 1 — Additional file 1. Interview Guide. [file 12913_2021_6184_MOESM1_ESM.docx]

| **Sub area** | **Key question(s)** | **Probes** |
| --- | --- | --- |
| **1.1 First meeting** | ***When did you first meet a Community Connectors?*** | Where was this?  Had you heard of them prior to your meeting?  Do you know roughly how many times you’ve met with a CCx?  ****If no direct contact – When/where did you see them?*** |
| **1.2 The process** | ***How did you meet them? (If not covered above)**** | Did they approach you? / Did you go to speak to them?  Did someone introduce them to you? If so, who was this?  Did they engage with your child?  ****If no direct contact - If you haven’t met them directly what is/was your experience of the CCx?*** |
| **1.3 Initial thoughts** | ***What were your initial thoughts about the Community Connector? **** | How did you feel about them approaching you? / Speaking to your child (use as appropriate)  Did they explain their role to you? What did this mean to you?  ****If no direct contact but as a group– How did they interact in the group? Did they explain their role? What do you think is the role of the CCx?*** |
| **1.4 Exploring the interaction** | ***Can you tell me a little more about the contact you you have had with the Connector?**** | How did you feel when you talked with them?  Was there anything they said that made you feel this way?  Do you feel they listened and/or understood?  Did you feel comfortable talking to them?  Did you have any contact outside of the children’s centre?  ****If no direct contact (i.e. in the room with a group) – In what settings have you seen them? Could you tell me more about this? What were they doing etc.?***  *Did you feel you could approach them? Was there a reason why you didn’t at that time?* |
| **1.5 Your relationship with CCx** | ***What do the CCx mean for you?*** | Did you have any expectations of them as a CCx?  Is this the same CCx or do you see different ones? – Could you name them?  How do you feel about this?  Would you have wanted more or less contact? |

**Area 1: To explore “primary” connections between the CCx and caregiver *- “We would like to start by talking about when you first met a connector…”***

**Area 2: (WHAT/WHERE) Secondary connections - *“We would like to talk more about activities you may have been directed to by the Community Connector…***

| **Sub area** | **Key question(s)** | **Probes** |
| --- | --- | --- |
| **2.1 Information /Signposting to activities** | **Did the CCx provide you with any information?** | Can you tell me a bit more about this?Did you find the information useful for your needs?  Did the CCx recommend any activities to you? Could you identify whether any of the following (use the full list of early years activities)  where recommended to you by the CCx? ****If no direct contact (i.e. in the room with a group) – Did the CCx provide any information to the group? Do you remember what this was? Was this useful?*** |
| **2.2 Types of activities** | **What types of activities did the CCx suggest?*** | Did CCx recommend any activities at the children’s centre?  Did CCx tell you about any other activities in the community for you as a parent?  Did CCx tell you about any other activities in the community for your child?  ****If no direct contact (or only as a group)- Did the CCx suggest any activities to the group?*** |
| **2.2 Awareness of activities** | ***Were you aware of these activities before speaking with the CCx?*** | Had you been to (X) before?  (If no) what was the reason for this?  Did you attend any of (X) because of the information given to you from CCx  (If relevant) What was it that the CCx said/did that encouraged you to attend?  Where the activities suggested relevant to you?  Why did you think that?  Is there any particular activity/or piece of information that stands out for you? |
| **2.3 Information provided by CCx** | ***Was there any other information that the CCx has spoken to you about?*** | Did the CCx tell you anything about child health and development?  Did you think this information was relevant to you ?Where there other areas you wanted to discuss with the Connector but didn’t?  What were these?  ****If no direct contact (i.e. in the room/event) – Would you have liked to have engagement with the CCx? – is there any information that you might like to speak to the CCx about?*** |
| **2.4 Connections** | ***Did the CCx talk about any other families you might speak with?*** | (If yes) Did they connect you to them?  (If yes) How did the CCx connect you to other families?  What did you think of this?Is this an area you think the CCx could help you with?  In what way?  Are you still in contact with the other family/ies |

**Area 3: Change - *“We would like to talk to you about any changes that may have happened for you and your child because of the CCx.***

| **Sub theme** | **Key question(s)** | **Probes** |
| --- | --- | --- |
| **3.1 Change** | ***Has the CCx made a difference to you / your child / family?*** | In what ways? Can you tell me a bit more about that?  Do you do anything differently because of this?  Could you describe how this has impacted you? |
| **3.2 Knowledge** | ***In what ways, do you think, has your knowledge of child development been impacted by the CCx?*** | Can you tell me a bit more about that?  How important is this for you? |
| **3.3 Participation** | ***Are there any other activities / groups that you have become involved in because of the CCx?*** | What was it the CCx said/did to encourage you to do (X)?  Do you feel more connected to your community?  Has the CCx changed the way you feel about other professionals? In what way? |
| **3.3 Continuation** | ***Do you continue to meet with the CCx?*** | What are/where your reasons for this?  Did you carry on practising (X)?  Did you tell anyone else about (X)? |

All caregivers will be thanked for their time and participation in the evaluation. They will be given the opportunity to express any views or opinions and to make any final comments or recommendations about any aspect of the CCx and any other topics discussed before the end of the interview.

**End of Interview**
